# Supplementary material for: Effectiveness and Safety of Ayurvedic Medicines in Type 2 Diabetes Mellitus Management: A Systematic Review and Meta-Analysis
Source: Front Pharmacol. 2022 Jun 8;13:821810. doi: 10.3389/fphar.2022.821810 (PMC9213670; doi:10.3389/fphar.2022.821810)
Supplement: Supplementary file 1 [file DataSheet1.zip › Supplementary Material/Forest and Funnel Plots/Punica granatum/Waist circumference.pdf]

Punica granatum - Waist circumference

Study	Intervention N	Intervention Mean	Intervention SD	Comparator N	Comparator Mean	Comparator SD	Mean Diff. with 95% CI	Weight (%)
Babaeian 2013	23	99.58	8.58	22	98.56	7.14	1.02 [-3.60, 5.64]	31.54
Faghihimani 2017	37	91.00	7.30	37	91.00	8.20	0.00 [-3.54, 3.54]	53.87
Grabež 2020	19	107.02	10.03	18	108.98	11.06	-1.96 [-8.76, 4.84]	14.59
**Overall**							0.04 [-2.56, 2.63]	

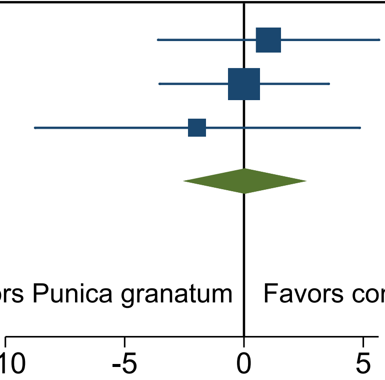

Heterogeneity: $\tau^2 = 0.00$, $I^2 = 0.00\%$, $H^2 = 1.00$

Test of $\theta_i = \theta_j$: $Q(2) = 0.51$, $p = 0.78$

Test of $\theta = 0$: $z = 0.03$, $p = 0.98$

Random-effects REML model
